# Root resorption during orthodontic treatment with Invisalign®: a radiometric study

**DOI:** 10.1186/s40510-017-0166-0

**Published:** 2017-05-15

**Authors:** Giulia Gay, Serena Ravera, Tommaso Castroflorio, Francesco Garino, Gabriele Rossini, Simone Parrini, Giovanni Cugliari, Andrea Deregibus

**Affiliations:** 1 Department of Surgical Sciences, University of Torino, Turin, Italy; 2 Department of Statistics and Quantitative Methods, University of Milano-Bicocca, Milan, Italy

**Keywords:** Adult patients, Aligners, Root resorption

## Abstract

**Background:**

Root resorption (RR) is described as a permanent loss of tooth structure from the root apex. Many reports in the literature indicate that orthodontically treated patients are more likely to have severe apical root shortening, interesting mostly maxillary, followed by mandibular incisors. The aim of the study was to investigate the incidence and severity of RR in adult patients treated with aligners. The study group consisted of 71 class I adult healthy patients (mean age 32.8 ± 12.7) treated with aligners (Invisalign®, Align Technologies, Santa Clara, CA, USA). All incisors, canines, upper first premolars, and first molars were assessed. Root and crown lengths of 1083 teeth were measured in panoramic radiographs at the beginning (T0) and at the end (T1) of clear aligner therapy. Individual root-crown ratio (RCR) of each tooth and therefore the relative changes of RCR (rRCR) were determined. A decrease of rRCR was assessed as a reduction of the root length during treatment.

**Results:**

All patients had a minimum of one teeth affected with a reduction of root length, on average 6.38 ± 2.28 teeth per patient. Forty one, 81% of the 1083, measured teeth presented a reduction of the pre-treatment root length. A reduction in percentage of >0% up to 10% was found in 25.94% (*n* = 281), a distinct reduction of >10% up to 20% in 12.18% (*n* = 132) of the sample. 3.69% (*n* = 40) of the teeth were affected with a considerable reduction (>20%).

**Conclusions:**

Orthodontic treatment with Invisalign® aligners could lead to RR. However, its incidence resulted to be very similar to that described for orthodontic light forces, with an average percentage of RR < 10% of the original root length.

## Background

Root resorption (RR) is a permanent loss of tooth structure from the root apex [1]. Its clinical outcomes in orthodontic patients are highly variable and depend on genetic predisposition, individual biologic variability, and mechanical factors [2]. Several authors demonstrated that RR occurs even without orthodontic treatment [3–6], but patients who underwent orthodontic treatment are more likely to show severe apical root shortening [7].

In histological studies, orthodontically moved teeth show an occurrence of RR greater than 90% [8–10]. Lower percentages are reported for diagnostic radiographic techniques. The average amount of tissue loss is less than 2.5 mm [11–14] or varies from 6 to 13% for different teeth [15] in radiographic studies.

RR is usually classified as minor or moderate in most orthodontic patients. Severe resorption, if exceeding 4 mm or one-third of the original root length, is seen in 1–5% of teeth^7^ [16–18].

Root resorption has two phases: during the first phase, the damage of the external surface of the root causes the exposition of denuded mineralized tissue, while in the second one, multinucleated cells are stimulated to colonize the denuded mineralized tissue, getting to a resorption process [19]. Without any further stimulation, cementum-like material will spontaneously repair the damage within 2–3 weeks. With persistent inflammatory process, deeper root dentin will be involved and RR radiographically detected [20]. When forces at the root apex exceed the resistance and reparative ability of the periapical tissues, RR occurs [21]. It begins approximately 2–5 weeks into treatment, but radiographical appearance requires 3–4 months.

Furthermore, the association between RR and the amount of orthodontic tooth movement^21^ [22–24] has been demonstrated. Since the amount of tooth movement depends on the severity of the malocclusion, a severe malocclusion represents a risk factor for RR. Class I patients with normal overjet show less RR than class II or III patients [25].

Several studies [26–28] suggest that light continuous forces are perceived as intermittent ones and allow the healing of the resorbed cementum, preventing further resorption. The Invisalign® treatment technique belongs to removable appliances, so intermittent forces are applied to the teeth. The aim of the present study was to investigate the incidence and severity of RR in adult patients treated with aligners.

## Methods

In the present study, we evaluated 71 (25 males and 46 females) adult healthy patients treated with aligners (Invisalign®, Align Technologies, Santa Clara, CA, USA). The mean age was 32.8 ± 12.7 (age range 18–71). We did not differentiate data by gender or age since previous studies pointed out that sex and age of patients could not be considered as potential confounding factors [29, 30]. In this prospective study, patients were recruited from December 2014 to December 2015 among the private practice patients in xxx, xxx. The panoramic radiographs were taken at the beginning (T0) and at the end (T1) of orthodontic treatment with the same device. The average treatment duration was 14 months.

Inclusion criteria for all the patients were adult patients (>18yo), normodivergent, and class I malocclusion with crowding (arch length discrepancy <6 mm).

Exclusion criteria were evidence of root resorption on pre-treatment panoramic radiographs, severely dilacerated roots, endodontically treated teeth, patients requiring other orthodontic systems, extraction therapy or any surgical treatment, and patients presenting tooth wear with dentin exposure at the initial examination.

The anterior crowding was resolved by IPR (interproximal enamel reduction) and/or protrusion of anterior teeth, determined by the orthodontist, depending on the initial overjet (protrusion) or crown’s shape (IPR). The mean IPR was 0.33 mm (min. 0 mm, max. 0.5 mm).

All incisors and canines, upper first premolars, and first molars were assessed. A total of 1083 teeth were evaluated.

The measurement of the dental panoramic radiographs was performed by using Orisceph® (Orisceph Rx®, Elite Computer Italia, Vimodrone, MI, Italia).

**Fig. 1:**
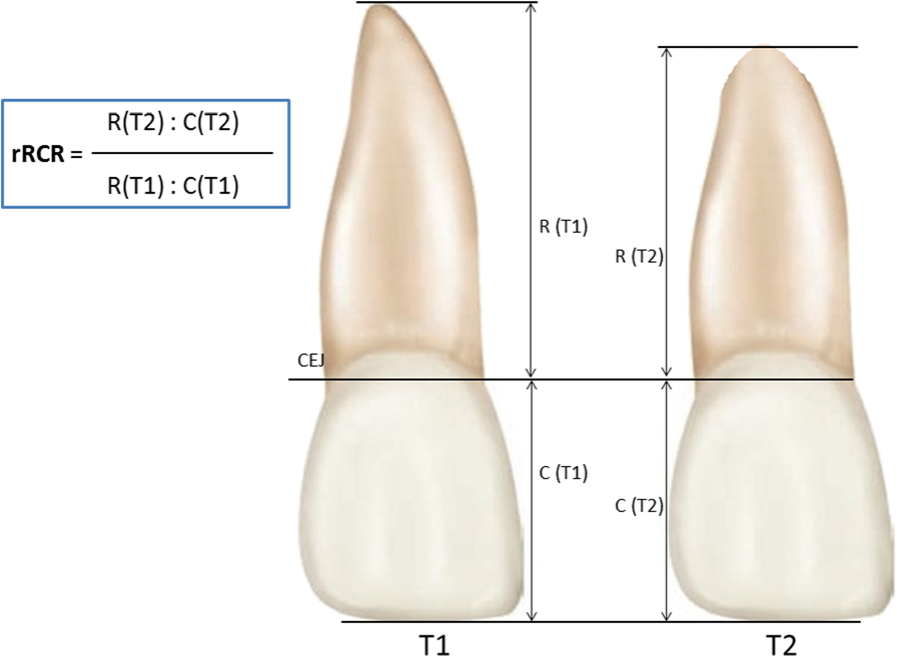
Measurement of the panoramic radiographs: root and crown lengths (CEJ = cemento-enamel junction). Individual root-crown-ratio (RCR) was determined considering pre- and post-treatment root and crown length

On the basis of Krieger et al. [31], Fritz et al. [32], and Linge and Linge^11^, all root and crown measurements were assessed by one examiner blinded about the study, in a stochastic sequence. The crown length was represented by the distance between incisal edge and cemento-enamel junction (on the long axis). The root length was represented by the distance between cemento-enamel junction and apex (Fig. 1).

As stated by Krieger et al. [31] and Fritz et al. [32], individual root-crown ratio (RCR) and therefore the relative changes of RCR (rRCR) were determined considering pre- and post-treatment root and crown length. An rRCR of 100% indicates no change of the pre-treatment root length relative to the post-treatment root length. A decrease of rRCR indicates a reduction of the root length during treatment.

Data analysis and collection were performed using the SPSS® software program (Statistical Package for Social Science) for Windows Version 23.0 (Inc., Chicago, II, USA).

The averages of the two measurements were used to calculate RCR and the changes in RCR. Absolute and relative frequencies of RCR were calculated for every tooth. Quantitative measurements are described by mean and standard deviation.

## Results

The mean rRCR for every tooth is shown in Table 1.

**Table 1:** Number of measured elements, mean, and standard deviation of RCR for every tooth

	1.6	1.4	1.3	1.2	1.1	2.1	2.2	2.3	2.4	2.6
No. of teeth	69	66	67	65	70	69	64	69	65	58
Mean rRCR (%)	100	103	104	104	102	103	100	104	102	101
Standard deviation	13.09	12.47	14.93	11.82	14.15	13.58	15.38	14.55	14.66	13.42
			4.3	4.2	4.1	3.1	3.2	3.3		
No. of teeth			71	71	70	70	68	71		
Mean rRCR (%)			107	102	100	106	104	105		
Standard deviation			13.78	16.5	13.28	13.48	12.27	13.48		

**Fig. 2:**
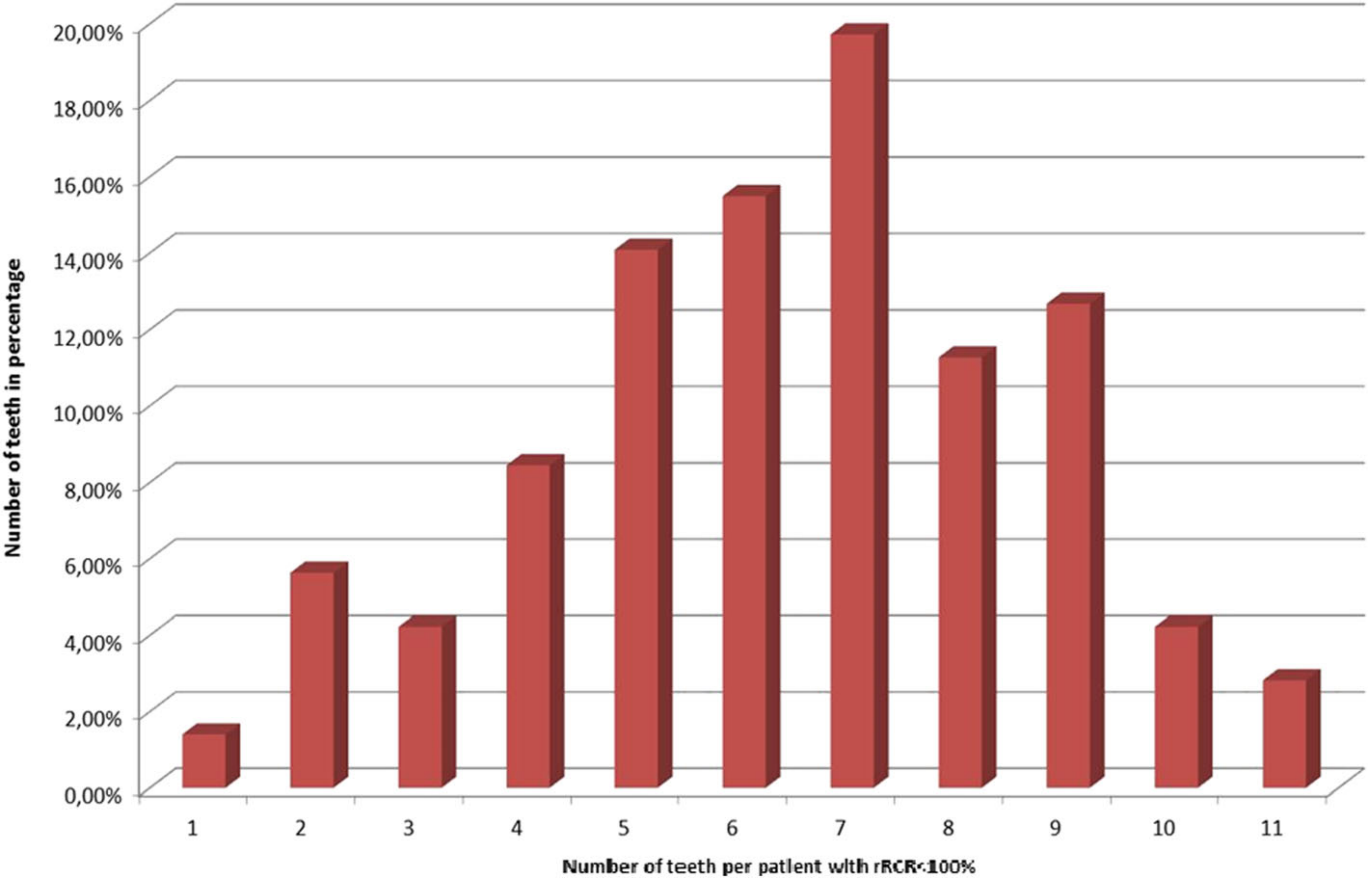
Distribution of the amount of affected teeth per patient

All patients had a minimum of one teeth affected with a reduction of the root length (rRCR < 100%), on average 6.38 ± 2.28 teeth per patient (Fig. 2).

Forty one percent (*n* = 29) of all patients had a minimum of one tooth with a 20% root length reduction.

In this study, 41.81% of the 1083 teeth (*n* = 453) showed a reduction of post-treatment root length (rRCR < 100%). A reduction up to 10% was found in 25.94% (*n* = 281), a distinct reduction from 10% up to 20% in 12.18% (*n* = 132) of the sample. 3.69% (*n* = 40) of the teeth were affected with a considerable reduction (>20%) (Table 2).

**Table 2:** Number and percentage of teeth presenting rRCR ≥ 100% (no RR), rRCR between 90 and 100 (slight RR), rRCR between 80 and 90 (moderate RR), rRCR ≤80 (severe RR)

rRCR (%)	No. of teeth	Percent
≥100	630	58.12
90 ≤ *X* < 100	281	25.94
80 ≤ *X* < 90	132	12.18
<80	40	3.69
TOT	1083	100

The values of the individual teeth are shown in Tables 3 and 4.

**Table 3:** Number and percentage of the upper teeth presenting rRCR = 100% (no RR), rRCR between 90 and 100 (slight RR), rRCR between 80 and 90 (moderate RR), and rRCR = 80 (severe RR)

RCR %	1.6	1.4	1.3	1.2	1.1	2.1	2.2	2.3	2.4	2.6
No. of teeth	36	39	41	46	39	39	31	41	36	27
≥100	52.17%	59.09%	61.19%	70.77%	55.71%	56.52%	48.44%	59.42%	55.38%	46.55%
No. of teeth	15	17	15	14	16	20	19	18	20	22
90 ≤ *x* < 100	21.74%	25.75%	22.39%	21.54%	28.86%	28.98%	29.69%	26.08%	30.77%	37.93%
No. of teeth	16	9	9	4	13	7	9	8	3	7
80 ≤ *x* < 90	23.19%	13.63%	13.43%	6.15%	18.57%	10.14%	14.06%	11.59%	4.61%	12.07%
No. of teeth	2	1	2	1	2	3	5	2	6	2
<80	2.9%	1.51%	2.98%	1.54%	2.86%	4.35%	7.81%	2.9%	9.23%	3.45%
TOT no. of teeth	69	66	67	65	70	69	64	69	65	58
%	100%	100%	100%	100%	100%	100%	100%	100%	100%	100%

**Table 4:** Number and percentage of the lower teeth presenting rRCR = 100% (no RR), rRCR between 90 and 100 (slight RR), rRCR between 80 and 90 (moderate RR), and rRCR = 80 (severe RR)

RCR %	4.3	4.2	4.1	3.1	3.2	3.3
No. of teeth	50	38	39	46	37	45
≥100	70.42%	53.52%	55.71%	65.71%	54.41%	63.38%
No. of teeth	15	17	12	17	25	19
90 ≤ *x* < 100	21.13%	23.94%	17.14%	24.28%	36.76%	26.76%
No. of teeth	6	10	13	7	5	6
80 ≤ *x* < 90	8.45%	14.08%	18.57%	10.00%	7.35%	8.45%
No. of teeth	0	6	6	0	1	1
<80	0.00%	8.45%	8.57%	0.00%	1.47%	1.41%
TOT no. of teeth	71	71	70	70	68	71
%	100%	100%	100%	100%	100%	100%

**Fig. 3:**
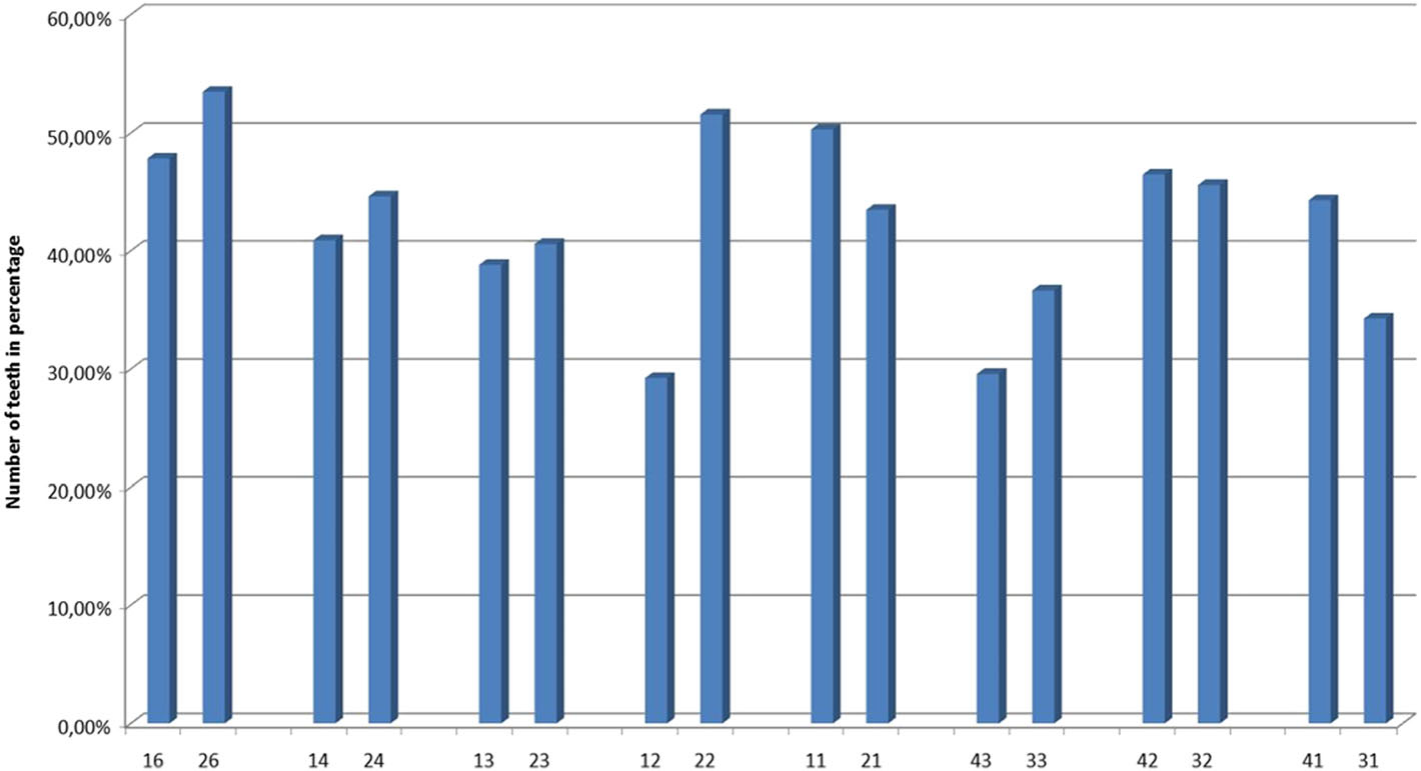
Percentage of teeth affected by RR (rRCR < 100%)

The percentage of teeth with rRCR < 100% are shown in the Fig. 3.

**Fig. 4:**
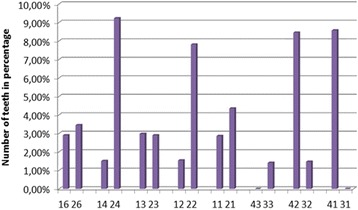
Percentage of teeth affected by severe RR (rRCR ≤ 80%)

A severe RR was observed only in 3.69% of teeth. As shown in Fig. 4, severe RR occurs mostly in the upper left premolars, upper left lateral incisors, lower right lateral, and central incisors.

## Discussion

A previous review from Rossini et al. [33] demonstrated that Invisalign® is effective for simple malocclusions treatment. Starting from this consideration, the present study investigated the incidence of RR in a sample of adult patients with class I malocclusions, showing a very limited incidence of significant severe RR.

As shown in Table 3, 41.81% of the 1083 analyzed teeth (*n* = 453) were affected by post-treatment reduction of the root length. Even if in the present study we did not investigate the direct comparison of aligner treatment outcomes with fixed conventional appliances ones, data reported by other studies recently investigating RR both with aligners and multibracket appliances [29] seem to be consistent. Lund et al. [34] reported an incidence of 91%, but crowding was resolved by multibraket appliances and first premolars extraction, with a resultant more complex treatment. Iglesias-Linares et al. [30] recently demonstrated that treatments with increased discrepancy index, due to sagittal apical displacement increase, were more likely associated with a higher incidence in RR. However, there were no statistically significant differences whether removable aligners or fixed appliances were used, when genetic predisposition is excluded.

When considering RR severity in our study, the incidence of minimal RR (<10%) was 26%, mild RR (10–20%) was 12%, and severe RR (>20%) was only 3.69%. These values are consistent with those reported by Krieger et al. [31] with a minimal RR ranged from 25 to 32%, mild RR from 11 to 18%, and severe RR from 1 to 14% for mandibular incisors.

In other studies [22–24, 32, 35–41], maxillary incisors showed a consistent average apical RR, more than any other analyzed tooth, followed by mandibular incisors and mandibular first molars.

Tieu et al. [42] in their systematic review evaluated RR in maxillary and mandibular incisors during non-surgical orthodontic treatment of class II division I malocclusions; as a result, the majority of teeth experienced mild to moderate resorption following treatment, and the prevalence of incisor root resorption ranged between 65.6 and 98.1%.

According to several authors (Weltman [1] Eisel [43] Elhaddaoui [44]) RR, measured on panoramic or periapical radiographs, is usually less than 2.5 mm, with a <20% percentage of severe resorption (>4 mm or >1/3 original root length) affecting mostly maxillary lateral incisors.

In the present study, the prevalence of severe RR in maxillary incisors ranged from 1.54% (12) to 7.81% (22) and in mandibular incisors from 0 (31) to 8.57% (41). These results are significantly lower than those described by the previous authors.

The higher incidence of RR in maxillary and mandibular incisors may be explained with the greater extending of movement of these teeth than the rest of dentition, and the root structure of the incisors, its relationship to bone and the periodontal membrane, which transfers most of the forces to the apex [30].

Schwartz et al. [45] suggested that an orthodontic force heavier than the partial pressure of the periodontal capillaries (26 g/cm^2^) lead to periodontal ischemia and consequently to RR. In their prospective study, Barbagallo et al. [46] quantify premolar cementum resorption generated by treatment with ClearSmile® (ClearSmile, Woollongong, Australia) aligners using x-ray microtomography. Comparing the obtained values with those of a fixed appliance generating heavy or light orthodontic forces, the results showed that the aligner group had a similar RR to the light-force group and approximately six times greater than the untreated control group. These findings could be explained by the finite element analysis conducted by Cattaneo et al. in 2009 [47] on the PDL performance under light force loading: light continuous forces are perceived as intermittent by the periodontium because of the viscoelastic nature of PDL and the application of vertical forces during function and parafunction.

Orthodontic treatment with Invisalign® aligners could lead to RR as any other orthodontic treatment. The incidence of RR resulted consistent to the one described for orthodontic light forces (RR < 10% of original root length). Further studies on more complex malocclusions treated with aligners are guaranteed in order to analyze RR incidence with respect to comprehensive orthodontic treatments.

## Conclusions

The present study investigated the incidence and severity of RR in adult patients treated with aligners during class I treatments. Every patient showed a minimum of one tooth with root length reduction. On average, 6.39 teeth per patient were affected. Overall, 41.81% of the measured 1083 teeth showed signs of apical root resorption, but only 3.69% a reduction of over 20% of the pre-treatment root length. Severe RR affected mostly the upper lateral incisors and lower lateral and central incisors.

## Abbreviations

IPR: Interproximal enamel reduction
RCR: Root-crown ratio
RR: Root resorption
rRCR: Relative changes of RCR
